# Supplementary material for: Angelica sinensis polysaccharide as potential protectants against recurrent spontaneous abortion: focus on autophagy regulation
Source: Front Med (Lausanne). 2025 Jan 15;12:1522503. doi: 10.3389/fmed.2025.1522503 (PMC11774876; doi:10.3389/fmed.2025.1522503)
Supplement: Supplementary material S1 — The main instruments used during the LC-MS process, along with their models/specifications and manufacturers. [file Supplementary_file_1.zip › Metabolomics sequencing data FC1.2/8.代谢通路分析/Exposure_Ctrl/all/KEGG/mmu00270.html]

KEGG PATHWAY: Cysteine and methionine metabolism - Mus musculus (house mouse)


# Cysteine and methionine metabolism - Mus musculus (house mouse)


[
Pathway menu
| Organism menu
| Pathway entry
| Show description
| Download
| Help
]

Cysteine and methionine are sulfur-containing amino acids. Cysteine is synthesized from serine through different pathways in different organism groups. In bacteria and plants, cysteine is converted from serine (via acetylserine) by transfer of hydrogen sulfide [MD:M00021]. In animals, methionine-derived homocysteine is used as sulfur source and its condensation product with serine (cystathionine) is converted to cysteine [MD:M00338]. Cysteine is metabolized to pyruvate in multiple routes. Methionine is an essential amino acid, which animals cannot synthesize. In bacteria and plants, methionine is synthesized from aspartate [MD:M00017]. S-Adenosylmethionine (SAM), synthesized from methionine and ATP, is a methyl group donor in many important transfer reactions including DNA methylation for regulation of gene expression. SAM may also be used to regenerate methionine in the methionine salvage pathway [MD:M00034].


##### Option

Scale:


100%

Image resolution:


 High

##### Search

##### ID search

##### Color

##### Module

Complete only
Including 1 block missing
Including any incomplete

- Pathway modules
  - Amino acid metabolism
    - Cysteine and methionine metabolism
      - M00021
        Cysteine biosynthesis
      - M00338
        Cysteine biosynthesis
      - M00609
        Cysteine biosynthesis
      - M00017
        Methionine biosynthesis
      - M00034
        Methionine salvage pathway
      - M00035
        Methionine degradation
      - M00368
        Ethylene biosynthesis


KGML

Image (png) file 1x
